# Validation of the German language version of the Chronic Ear Survey and its psychometric comparison with an established German measurement instrument

**DOI:** 10.1007/s00106-023-01335-5

**Published:** 2023-10-04

**Authors:** Michael Knoke, Marcus Neudert, Thomas Zahnert, Susen Lailach

**Affiliations:** 1 Klinik für Hals‑, Nasen‑, Ohrenheilkunde, Charité—Universitätsmedizin Berlin, Campus Virchow-Klinikum, Campus Charite Mitte, Augustenburger Platz 1, 13353 Berlin, Germany; 2 Universitätsklinikum Carl Gustav Carus Dresden, Dresden, Germany

**Keywords:** Quality of life, Questionnaire, Otitis media, Epidemiologic measurements, Middle ear

## Abstract

**Background:**

With the Chronic Ear Survey (CES), a validated measurement instrument for the assessment of disease-specific health-related quality of life (HRQoL) has been available internationally since 2000. The aim of this study was to provide a validated German version of this international instrument and to compare it with the German Chronic Otitis Media Outcome Test 15 (COMOT-15).

**Methodology:**

The CES was translated into German via a forward-backward translation process. For validation, 79 patients with COM undergoing middle ear surgery were prospectively included. HRQoL was determined preoperatively and 6 months postoperatively using the CES and the COMOT-15. Pure tone audiometry was also performed at both measurement time points. In the control examination, an additional retrospective assessment of the preoperative situation was additionally performed using the CES and the COMOT-15 to assess the response shift. The determined psychometric characteristics were internal consistency, test–retest reliability, discrimination validity, agreement validity, responsiveness, and response shift for both measurement instruments. Convergent validity of both measurement instruments was assessed using linear regression.

**Results:**

On the basis of the CES, patients with COM could be reliably distinguished from patients with healthy ears. The CES showed satisfactory reliability with high internal consistency (Cronbach α 0.65–0.85) and high retest reliability (r > 0.8). The global assessment of HRQoL impairment correlated very well with the scores of the CES (r = 0.51). In addition, it showed a high sensitivity to change (standardized response mean −0.86). Compared to the COMOT-15, it showed a lower response shift (effect size −0.17 vs. 0.44). Both measurement instruments correlated only slightly with air conduction hearing threshold (r = 0.29 and r = 0.24, respectively). The concordant validity of both measurement instruments was high (r = 0.68).

**Conclusion:**

The German version of the CES shows satisfactory psychometric characteristics, so that its use can be recommended. The CES focuses on the influence of ear symptoms on HRQoL, whereas the COMOT-15 also includes functional and psychological aspects. Due to only minor response shift effects, the CES is particularly suitable for studies with multiple repeat measurements.

**Supplementary Information:**

The online version of this article (10.1007/s00106-023-01335-5) contains the original Chronic Ear Survey in English.

Chronic otitis media (COM) is a disease characterized by a combination of different symptoms such as hearing loss, otorrhea, otalgia, headache, but also tinnitus. In particular, the impairment of hearing function with the associated reduced ability to communicate can lead to social withdrawal, dejection [18], and thus a reduction in the disease-specific quality of life (health-related quality of life, HRQoL; [11]).

The HRQoL as a dimension of outcome quality in patients with chronic otitis media unfortunately still plays a subordinate role in German-speaking countries. Therapy quality continues to be measured mainly by audiometric parameters and healing or recurrence rates. Fortunately, HRQoL has become increasingly important in the past 15 years [12].

Initially, HRQoL was also assessed in middle ear surgery preferably using generic measurement instruments, such as the Short Form 36 (SF-36), although no treatment effect could be demonstrated using the generic HRQoL measurement, as these measurement instruments do not adequately reflect the symptomatology of COM and its effects [2, 13, 16]. Accordingly, the efforts of the otological community to increasingly provide disease-specific measurement instruments for COM are understandable and to be welcomed. In the meantime, there are six international measurement instruments for assessing HRQoL in patients with COM, which have been validated in different languages and differ in the dimensions they cover [12]. In 2000, Nadol et al. provided the first disease-specific HRQoL measurement tool for patients with COM in English, the Chronic Ear Survey (CES; [16, 25]). Italian, Dutch, and Chinese validated versions of this measurement instrument are now also available [7, 21, 25]. In German-speaking countries, the Zurich Middle Ear Inventory 21 (ZCMEI-21) and the Chronic Otitis Media Outcome Test 15 (COMOT-15), two measurement instruments with divergent focuses, are used [1, 2]. Due to the existing national and international heterogeneity of HRQoL measurement instruments, a comparative evaluation of HRQoL studies is hardly possible, especially since to date there are no international and national recommendations for the selection of a measurement instrument [8]. Finally, the situation is complicated by the provision of proprietary translation versions of existing measurement instruments without appropriate validation of the measurement instrument. A translation of a measurement instrument requires more than a simple translation. For any translation, a validation process is necessary to examine whether the measurement instrument actually measures what it claims to measure.

In order to evaluate the heterogeneity of the measurement tools, a comparative study was recently conducted to assess the COMOT-15 and ZCMEI-21 in German. Both measurement tools showed a high concurrent validity, so that a conversion equation for estimating the respective total scores of both measurement tools could be provided [14].

The aim of this study was therefore first to provide a validated German-language version of the internationally used CES. Furthermore, a comparison of its psychometric characteristics with the COMOT-15 established in the German-speaking area was carried out in order to derive recommendations for the goal-oriented application of patient-reported outcome measures (PROMs) in everyday clinical practice and in clinical studies.

## Patients and methods

The study was approved by the ethics committee of the University Hospital Dresden (EK 268072014).

A total of 79 patients who underwent COM surgery at Dresden University Hospital between May 2016 and May 2018 were prospectively recruited. Patients who were under 18 years of age and not legally competent were excluded, as were patients who had to undergo another ear surgery during the study period.

All patients received a pure-tone audiogram preoperatively and for a control 6 months postoperatively. To evaluate the audiological data, the 4‑frequency pure-tone average value of the air conduction threshold and the air–bone gap (ABG) was averaged over the frequencies 0.5, 1, 2, and 4 kHz. At both time points, HRQoL was assessed using the German-language version of the CES and the COMOT-15 questionnaire, which is already validated in German. A further assessment of quality of life using CES and COMOT-15 was performed 1 week after the follow-up examination.

As a control group, ear-healthy patients with subjective normal hearing were included after being informed about the study project and the study procedure.

### Chronic Ear Survey

The CES is a psychometric measurement tool that uses 13 items to assess the frequency, duration, and severity of symptoms associated with COM, and thus the disease-specific HRQoL [16]. The answers to each question vary from frequency ratings to Likert-scale 4‑ to 6‑point response options. The measurement instrument is divided into three subscores, “Limitations in Activities” (questions a1–a3), “Symptoms” (questions s1–s7), and “Use of Medical Resources” (questions m1–m3). Each question is transformed to a scale from 0 to 100. To calculate the total score, the values of the subscores must be divided by the number of questions included. Afterwards, the subscores are added and divided by 3 to get the total CES score. Higher scores are associated with a better quality of life.

### Chronic Otitis Media Outcome Test 15

The COMOT-15 was developed and validated as a disease-specific measurement tool to assess HRQoL in patients with COM primarily in the German language [2]. Based on 15 items, which are 5‑level Likert-scaled, three subscores are formed in addition to a total score (items 1–13): “Ear Symptoms” (items 1–6), “Hearing Function” (items 7–9), and “Mental Health” (items 10–13). In addition, there is a general assessment of disease-specific HRQOL (item 14) and an indication of the frequency of medical visits, “Medical Resource Utilization” (item 15). To evaluate the COMOT-15, the score is transformed to a scale from 0 to 100, with a value of 0 representing the least restrictive HRQOL.

### Validation of the German-language CES

Translation of the CES from English into German was performed by two native-speaker professional translators. The consistency between these translations was checked and no deviations from the content of the questionnaire were found. Afterwards, two native-speaker translators translated the questionnaire back into English. Both versions of the questionnaire were reviewed by two otologists to confirm that the questions were understandable and that the original meaning was maintained.

**Fig. 1:**
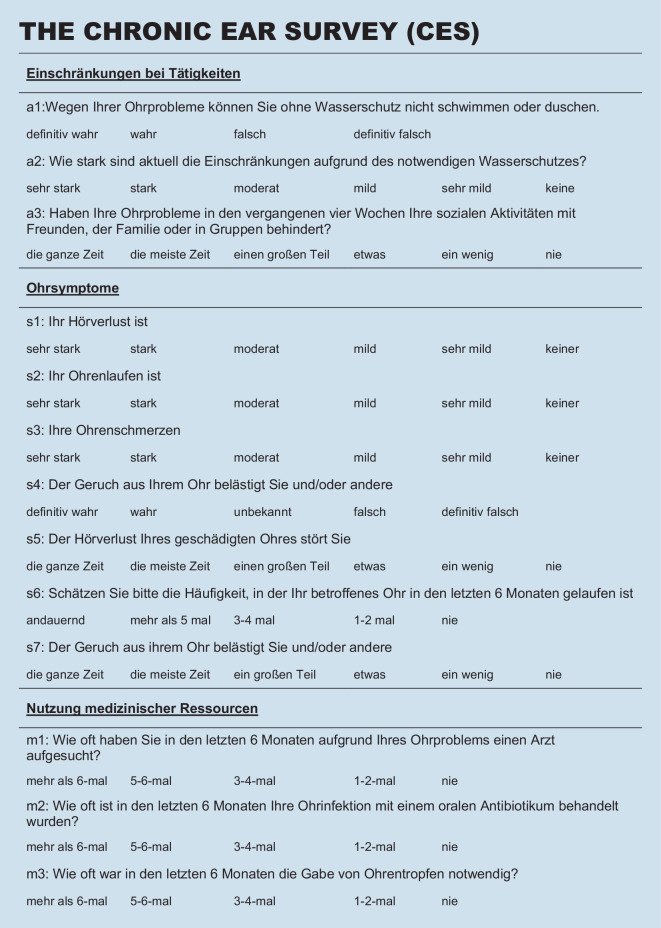
Chronic Ear Survey (*CES*) in German

To validate the German version of the CES (Fig. 1), the reliability, validity, and responsiveness of the measurement tool were examined.

### Response Shift

In addition, a possible response shift was investigated. This refers to a change in the assessment background of the HRQoL due to a critical event [22]. A response shift occurs primarily when people confront critical and threatening life events [23]. Such events are not only found in oncology; they can also include the disclosure of diagnoses or the start of treatment in the context of a chronic disease. A response shift must be taken into account in the context of longitudinal measurements. The larger the response shift between two measurement points, the smaller the difference values of the longitudinal measurement to be considered as a true quantitative change in quality of life. However, biases due to faulty recall in the context of then-testing should also be taken into account. [19]. To determine the response shift, an additional retrospective assessment of HRQoL by patients using the CES and the COMOT-15 was made during the control presentation.

### Statistical evaluation

For statistical analysis, PASW Statistics 25.0 (SPSS Inc., Chicago, IL, USA) was used. The distribution properties are described by arithmetic mean and standard deviation.

The mean, range, variance, and discriminatory power of the respective items were determined within the framework of the *single-item analysis* of the CES. Item difficulty was assessed using a difficulty index by forming the quotient of item mean and maximum achievable score in an item multiplied by 100. Values from 15 to 85 are recommended as item difficulty [6]. Items with an item difficulty < 15 were considered too difficult, and those with a difficulty > 85 were considered too easy. Cut-off values of 0.3 or more were considered acceptable [15].

To assess *reliability*, the internal consistency of the measurement tool was first tested by Cronbach’s α for the total score and the subscores. Psychometric instruments should only be used if Cronbach’s α reaches a value of ≥ 0.65 [24]. Furthermore, the test–retest reliability was checked. Therefore, all patients were asked to answer the questionnaire twice postoperatively: at the time of the follow-up consultation and 1 week after the follow-up consultation. The results of the CES at these two measurement times were evaluated using a correlation analysis (according to Pearson). Correlation coefficients of 0.70–0.95 are considered high to very high [20].

To determine the *validity* of the measurement tool, the concurrent validity was assessed by determining Pearson’s correlation coefficient for the total score and the subscores with a global disease-specific question (question 14: overall assessment of the impairment of quality of life due to the ear disease) from the already validated COMOT-15 questionnaire. Discriminant validity was determined by comparison with an “ear-healthy” control group (patients without COM) using a *t* test.

*Sensitivity to change* was described through the standardized response mean (SRM), which is defined as the quotient of the mean change and the standard deviation of the change. Values of ≥ 0.8 are thereby assessed as a large effect; ≥ 0.5, < 0.8 as a medium effect; ≥ 0.2, < 0.5 as a small effect and < 0.2 as a minor effect [4].

A possible *response shift* was tested using a paired *t* test in the sense of an indirect change measurement. At the second measurement point, a retrospective assessment of the first measurement point is made (then-test). The standardized effect size (SES) is calculated to estimate the size of the effect. In the effect size calculation, it is fundamental that the mean difference from the two measurement points is relativized to a measure of dispersion [9]. For SES, the standard deviation of the measurement preoperatively is used for this purpose. For the evaluation of effect sizes, the classification according to Cohen has become established [4]. Effects of approximately 0.2 are described as small, approx. 0.5 as medium, and approx. 0.8 as large. Additionally, effect sizes of ≥ 0.5 are considered clinically relevant [5, 19].

The relationship between the total CES and COMOT-15 scores was modeled using linear regression. The significance level was defined as *p* < 0.05.

## Results

### Patient characteristics

The study included 79 patients with COM. The demographic parameters of this patient group are shown in Table 1.

**Table 1:** Demographic data of the patient group

		Preoperative patient data (*n* = 79)
Age		48.6 ± 14.9 (SD) years
Gender	Female	50 (63%)
	Male	29 (37%)
Pathology	Chronic suppurative otitis media	41 (52%)
	Cholesteatoma	38 (48%)
Hearing function	Air duct (0.5–4 kHz)	48.09 ± 20.05 dB
	ABG (0.5–4 kHz)	24.25 ± 14.49 dB

*ABG* air–bone gap, *SD* standard deviation

At the follow-up examination 6 months postoperatively, data of 59 patients were collected (response rate: 75%). Of these patients, 30 had cholesteatoma and 29 had a chronic suppurative otitis media (mucosal disease).

The ear-healthy control group included 30 individuals with a mean age of 30.9 ± 8.1 years. The gender distribution was comparable to the patient group with 18 females (60%) and 12 males (40%; *p* = 0.82). The mean age was younger than in the group with COM (30.9 ± 8.1 vs. 48.6 ± 14.9 years; *p* < 0.001).

### Psychometric characteristics of the German-language CES

The CES showed acceptable internal consistency in the overall score and subscores in the German-language version pre- and postoperatively (Table 2).

**Table 2:** Psychometric characteristics of the German-language CES and the COMOT-15 compared with the respective original publication

	Cronbach’s α, preoperative		Cronbach’s α, postoperative		Test–retest reliability (*r*)		Concurrent validity (*r*)		SRM		Correlation (*r*) with the air conduction threshold (0.5–4 kHz)		Response shift (SES)
	Own data	Original	Own data	Original	Own data	Original	Own data	Original	Own data	Original	Own data	Original	Own data
*CES*													
Total score	0.85	0.83	0.83	–	0.89***	–	0.51***	–	−0.86	0.42	0.29*	0.15	−0.17
Limitations in activities	0.65	0.62	0.68	–	0.84***	–	0.41***	–	−0.50	–	0.20	−0.11	−0.21
Symptoms	0.84	0.80	0.76	–	0.82***	–	0.51***	–	−0.71	–	0.33**	0.33*	−0.07
Use of medical resources	0.77	0.75	0.77	–	0.92***	–	0.13	–	−0.64	–	0.10	0.18	−0.19
*COMOT-15*													
Total score	0.88	0.89	0.88	0.89	0.90***	0.89	0.78***	0.72***	0.79	0.44	0.24	–	0.44
Ear symptoms	0.79	0.91	0.79	0.91	0.85***	0.83	0.59***	0.56***	0.69	0.46	0.08	–	−0.20
Hearing function	0.90	0.91	0.91	0.90	0.89***	0.81	0.64***	0.56***	0.46	0.24	0.34**	–	0.59
Mental health	0.87	0.90	0.88	0.90	0.82***	0.86	0.73***	0.69***	0.70	0.32	0.19	–	0.37

The psychometric data of the original studies for the COMOT-15 are taken from the publication by Baumann et al. 2009 [2] and for the CES from the publication by Wang et al. 2000 [25]

“–” no data available, *SES* effect size, *SRM* standardized response mean, *CES* Chronic Ear Survey, *COMOT-15* Chronic Otitis Media Outcome Test 15

*p ≤ 0.05 significant, **p < 0.01 very significant, **p ≤ 0.001 highly significant

When test–retest reliability was determined, correlation coefficients of > 0.80 were obtained for the total score as well as for all subscores, implying high test–retest reliability (Table 2).

Concurrent validity was tested by correlating the global health-specific question (Question 14: Overall assessment of impairment of quality of life due to ear disease) of the COMOT-15 with the total score and subscores of the CES. This showed a positive correlation for both the total score and the subscores “Limitation in Activities” and “Symptoms” (Table 2). The medical resource use subscore showed no significant correlation.

**Fig. 2:**
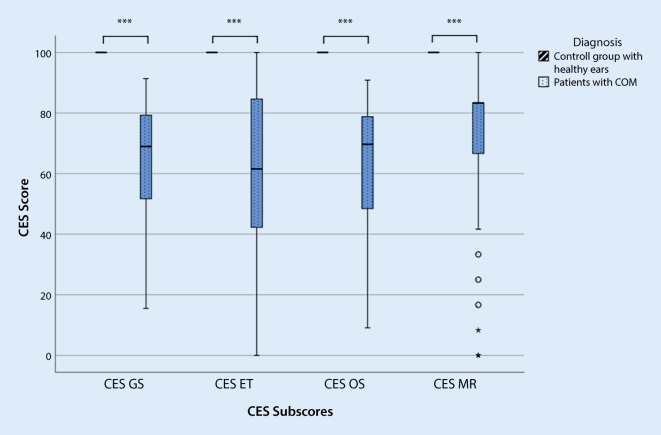
Testing the discriminant validity of the Chronic Ear Survey (*CES*) between patients’ preoperative data (*n* = 79) and a group of ear-healthy individuals (*n* = 30). *CES GS* CES Total score, *CES ET* CES Subscale activity restriction, *CES OS* CES Subscale symptom,* CES MR* CES Subscale medical resource utilization, *COM* chronic otitis media. ***p ≤ 0.001 highly significant

To investigate discrimination ability, the German translation of the CES was also completed by an ear-healthy control group (*n* = 30). In all scales, the control group reported a significantly lower impairment in HRQoL than the patient group COM (Fig. 2).

**Fig. 3:**
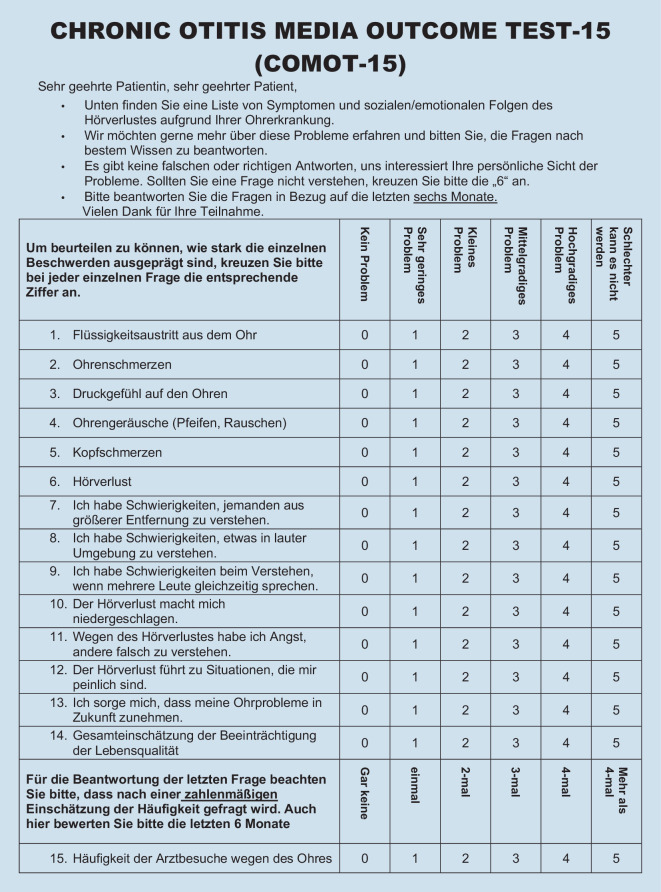
Chronic Otitis Media Outcome Test 15 (*COMOT-15*)

### Comparison of the German language CES with the COMOT-15

Evaluation of the item difficulty (Table 3) of the individual items of the CES showed that they were within the recommended limits with the exception of item m3 (Frequency of oral antibiotic administration, item difficulty 85.75). For the COMOT-15, the item difficulty of all items was within the recommended cut-off range. While the CES included mainly questions of medium-to-easy difficulty, the items of the COMOT-15 had medium-to-high difficulty. The discriminatory power for all items of the CES and the COMOT-15 was > 0.3, which was acceptable. Responses covered the maximum range of each item for all items.

**Table 3:** Indication of variance, discriminatory power, and item difficulty for the items of the CES and the COMOT-15

Item	MW	SD	Variance	Discriminatory power	Item difficulty	Range
*CES *(Fig. 1)						
a1	1.48	1.12	1.25	0.42	49.33	0–3
a2	3.09	1.76	3.08	0.49	64.8	0–5
a3	3.23	1.55	2.41	0.43	64.6	0–5
s1	1.75	1.19	1.42	0.47	35	0–5
s2	3.68	1.64	2.68	0.67	73.6	0–5
s3	3.84	1.35	1.83	0.53	76.8	0–5
s4	2.61	1.34	1.81	0.65	65.25	0–4
s5	2.06	1.39	1.93	0.47	41.2	0–5
s6	2.86	1.44	2.07	0.64	71.5	0–4
s7	4.01	1.44	2.06	0.68	80.2	0–5
m1	2.1	1.14	1.3	0.46	52.5	0–4
m2	3.43	0.89	0.79	0.5	85.75	0–4
m3	3.25	1.14	1.29	0.39	81.25	0–4
*COMOT-15 *(Fig. 3)						
1	1.33	1.56	0.43	0.45	26.6	0–5
2	1.27	1.25	1.56	0.47	25.4	0–5
3	1.38	1.41	1.98	0.33	27.6	0–5
4	2.09	1.68	2.83	0.41	41.8	0–5
5	1.19	1.58	2.49	0.5	23.8	0–5
6	3.03	1.27	1.62	0.65	60.6	0–5
7	3.27	1.14	1.3	0.68	65.4	0–5
8	3.29	1.16	1.34	0.73	65.8	0–5
9	3.22	1.23	1.5	0.64	64.4	0–5
10	2.01	1.55	2.4	0.76	40.2	0–5
11	2.39	1.57	2.47	0.76	47.8	0–5
12	2.2	1.65	2.73	0.7	44	0–5
13	3.09	1.2	1.44	0.48	61.8	0–5
14	2.84	1.27	1.6	0.77	56.8	0–5
15	2.95	1.42	2.02	0.3	59	0–5

*CES* Chronic Ear Survey, *COMOT-15* Chronic Otitis Media Outcome Test 15, *MW* mean value, *SD* standard deviation

**Fig. 4:**
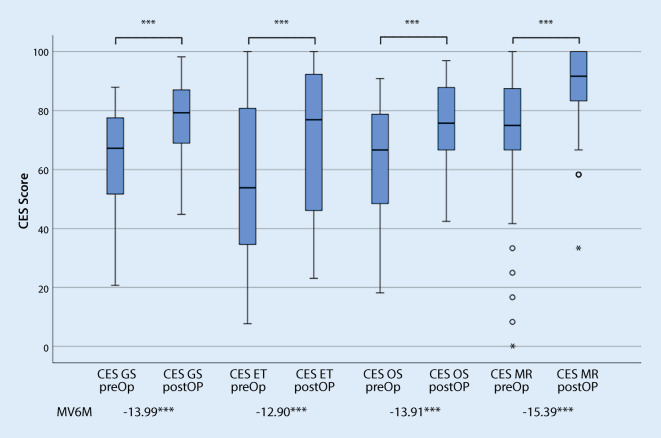
Responsiveness of the Chronic Ear Survey (*CES*): pre- and postoperative comparison (*n* = 59). *CES GS* CES Total score, *CES ET* CES Subscale activity restriction,* CES OS* CES Subscale symptom,* CES MR* CES Subscale medical resource utilization,* MV6M* Mean difference of scores of CES preoperatively—6 months postoperatively. ***p ≤ 0.001 highly significant

**Fig. 5:**
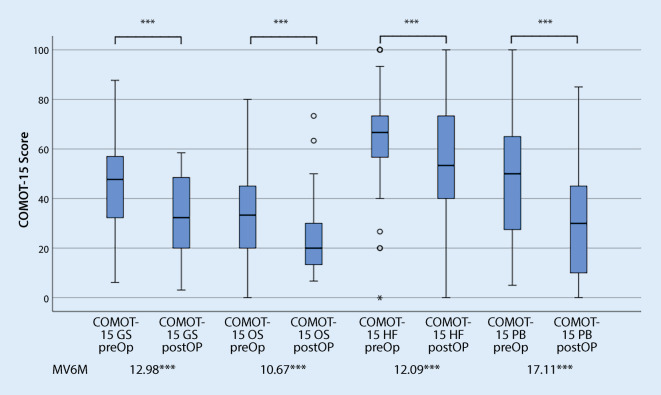
Responsiveness of the Chronic Otitis Media Outcome Test 15 (*COMOT-15*): pre- and postoperative comparison (*n* = 59). *COMOT-15 GS* COMOT-15 Total score, *COMOT-15 OS* COMOT-15 Subscale ear symptoms,* COMOT-15 HF* COMOT-15 Subscale hearing function, *COMOT-15 PB* COMOT-15 Subscale mental health,* MV6M* Mean difference of scores of CES preoperatively—6 months postoperatively. ***p ≤ 0.001 highly significant

In the pre- and postoperative comparison, the use of both the CES and the COMOT-15 showed a significant improvement in HRQoL in the total score and all subscores (Figs. 4 and 5). In accordance with Cohen’s proposed evaluation, a high effect was demonstrated when using the CES and a moderate effect when using the COMOT-15. Looking at the subscores of both measurement tools, a medium effect can be presented for all subscores. Only for the subscore “Auditory Function” of the COMOT-15 was the responsiveness small.

For both measurement tools, there was only a poor correlation of the postoperative total scores with the postoperative air conduction threshold. Only the subscores “Symptoms” of the CES and “Hearing Function” of the COMOT-15 correlated moderately with the air conduction threshold (Table 2).

The CES and the COMT-15 correlate strongly in the total score (*r* = 0.68, 95% confidence interval [CI]: 0.55–0.78). The “Symptoms” subscore of both measurement tools (*r* = 0.67, 95% CI: 0.53–0.77) and the “Medical Resource Utilization” subscores (*r* = 0.69, 95% CI: 0.34–0.9) also showed a strong correlation.

Using linear regression to model the relationship between CES and COMOT-15 total scores resulted in the following equations:

$$\text{Total score CES}=-0.56\times \text{Total score COMOT}\mathrm{-}15+96.36$$

$$\text{Total score COMOT}\mathrm{-}15=-0.68\times \text{Total score CES}+89.57$$

To test for a possible response shift, a paired *t* test was performed between the respective total scores of the preoperative survey and the postoperative assessment of the preoperative situation. The total scores of the CES and the COMOT-15 all showed a shift toward better quality of life (Table 2). The effect size (SES) showed a small effect for the CES (SES = −0.17). By contrast, the COMOT-15 (SES = 0.44) showed a medium effect. In the subscore analysis, a relevant positive response shift bias was found for the subscore “Auditory Function” of the COMOT-15 with an SES of 0.59. The subscore “Psychological Well-Being” (SES = 0.37) showed a medium effect. Only the subscore “Ear Symptoms” (SES = −0.2) of the COMOT-15 showed a negative response shift bias, but this must be classified as a small effect. Overall, the analysis of the CES showed small-to-negligible effects. The negative values in the effect size are also to be evaluated here as a positive shift, since with the CES higher scores are associated with a better quality of life, whereas with the COMOT-15 a higher score is equivalent to a lower HRQoL.

## Discussion

Assuming that the objective parameters used reflect the outcome of a surgical intervention inadequately, additional evidence of improvement in HRQoL has long been required [10]. There are a number of measurement tools available in the Anglo-American world for measuring HRQoL in patients with COM, for which there is no validated counterpart in the German-speaking area [12]. This results in a lack of comparability of international data [17].

The present German translation of the CES showed a high internal consistency. The Cronbach α values pre- and postoperatively (0.65–0.85) were higher than the values of the English version validation of the CES (0.60–0.83; [25]). The evaluation of the test–retest reliability showed Pearson correlation coefficients of > 0.8 for all subscores and the total score, which indicates a high reliability of the German version of the CES and is in agreement with the data of the original publication [25]. Furthermore, it was shown that the present version of the CES is able to distinguish well between patients with COM and ear-healthy patients. In addition, the CES shows good agreement with the patients’ overall assessment in correlation with a global health-specific question.

While the individual items of the COMOT-15 were developed on the basis of an extensive single-item analysis from an initial version of the measurement instrument comprising 31 items [2], no evaluation of the individual items was performed during the validation of the CES [16]. The items of the CES were selected on the basis of the content assessment of a group of experts. Despite this possible methodological weakness of the CES, the individual items of the German-language version of the CES also had satisfactory distribution characteristics overall. Only one item (item m2, Recording of oral antibiotic administration) was too easy for the patients, but had good discriminatory power. For the COMOT-15, good distribution parameters were demonstrated for all items, analogous to the validation study.

In terms of responsiveness, there was a medium effect for the subscores and even a large effect for the total score of the CES, whereas only moderate effects were detected when using the original English-language version [25]. Compared to the COMOT-15, a higher sensitivity to change was found for the total score of the CES. For both measurement tools, the highest responsivity was found for the subscores “Ear Symptoms.” Due to the content of its items, the CES focuses strongly on the expression of ear symptoms, which contributes to its higher sensitivity to change in the total score. Compared to the COMOT-15, the CES proved to be significantly more robust against response shift effects in the total score as well as in the subscore “Ear Symptoms.” These properties make the German-language CES particularly useful for repeated measurements of HRQoL to assess treatment effects over time, when the focus is on determining subjective impairment due to ear symptoms.

The COMOT-15 also showed a high sensitivity to change. However, in contrast to the CES, more pronounced response shift effects were observed, which even reached a clinically relevant level for the subscore “Hearing Function.” To date, there are hardly any studies on the significance of a response shift for the evaluation of HRQoL results in patients with COM. A response shift can be expected when people deal with critical and threatening life events [23]. Such events are most commonly found in oncology and chronic disease. Knowledge of the influence of a response shift is particularly crucial for the evaluation of the longitudinal measurements. The larger the response shift between two measurement time points, the smaller the difference values of the longitudinal measurement to be interpreted as a true quantitative change in HRQoL. On the other hand, possible biases due to faulty recall must also be taken into account [19]. For the other measurement tools available to determine HRQoL in COM, the response shift has not yet been determined.

The highest response shift was found for the “Hearing Function” subscore of the COMOT-15. Compared to the other currently available HRQoL measurement tools, the COMOT-15 targets hearing function and its effects on HRQoL most strongly. Nevertheless, the total score of the measurement tool, but also its subscore “Hearing Function” show only a poor correlation with the determined air conduction threshold. Due to the dominance of hearing-related items, a stronger correlation of the air conduction hearing curve with the COMOT-15 compared with the CES was to be expected. Surprisingly, the determined correlation coefficients for the total scores of both measurement tools did not differ. When looking at the CES, the highest correlation coefficient was found for the subscore “Ear Symptoms.” This subscore, however, did not correlate with the air conduction hearing threshold in the COMOT-15. This discrepancy can be explained by the fact that the subscore “Ear Symptoms” of the CES, in contrast to the COMOT-15, includes hearing loss as a symptom. Therefore, it can be deduced for the user that the COMOT-15 should be preferred over the CES for studies in which subjective hearing function and its effect on HRQoL are to be analyzed. However, for intervention studies and repeated measurement time points, the high response shift must then be considered for the interpretation of the results.

The low correlation of both measurement tools with hearing function suggests that other patient-associated characteristics also influence the disease-specific HRQoL. In a clinical study, the COMOT-15 was recently shown to be significantly influenced by patient depression, even after adjusting for other possible factors such as hearing function, level of pathology, and individual comorbidities [13]. For the CES, gender effects, diabetes mellitus, postoperative complications, educational level, and surgical technique were shown to influence the total score [3].

In contrast to the COMOT-15, the CES covers the “Use of Medical Resources” more comprehensively. Whereas the COMOT-15 considers this aspect with only one single item for recording the frequency of physician visits, this domain is recorded in the CES based on three items in an independent subscore. Despite the low number of items, the validation of the German version showed a high internal consistency for this subscore. Fortunately, there was a strong correlation between the result of the individual item on resource utilization of the COMOT-15 and the subscore of the CES, so that the recording of medical resource utilization in the COMOT-15 with only one item can also be classified as appropriate.

The COMOT-15 showed a stronger correlation (*r* = 0.78) of the total score with a global question on the patients’ overall assessment of HRQoL compared to the CES (*r* = 0.51). Since the CES focuses strongly on the expression of symptoms, the HRQoL is only indirectly reflected in the subscores “Limitations in Activities” and “Ear Symptoms” in this measurement tool. By contrast, the COMOT-15 contains an independent subscore for the assessment of psychological well-being, which showed the strongest correlation of all subscores with the global question (*r* = 0.73). Against this background an application of the COMOT-15 is to be preferred, if the psychosocial dimension in particular of the HRQoL is to be elaborated.

Overall, the similar content of the CES and COMOT-15 results in a significant correlation of the total scores and the concordant subscores of “Symptoms” and “Medical Resource Utilization.” This high concurrent validity may allow for conversion of both measurement tools to compare data between different centers or studies. However, this modeled relationship of both measurement tools should not replace a joint application of the two measurement tools.

## Practical conclusion

- The German version of the Chronic Ear Survey (CES) has satisfactory psychometric characteristics, so that its use can be recommended.
- Due to its high sensitivity to change, the CES is particularly suitable for intervention studies to determine pre- and postoperative health-related quality of life (HRQoL).
- In contrast to the Chronic Otitis Media Outcome Test 15 (COMOT-15), the CES shows only a small response shift, so that it is particularly suitable for investigations with several repeat measurements.
- On the basis of the high concordant validity, the use of both measurement tools (CES and COMOT-15) can be recommended.

### Supplementary Information





## Declarations

The study was reviewed and approved by the Ethics Committee of the Medical Faculty Dresden (EK 268072014). All described studies in humans were performed with the approval of the responsible ethics committee, in accordance with national law and the Declaration of Helsinki of 1975 (in the current, revised version). Informed consent was obtained from all patients involved.

The supplement containing this article is not sponsored by industry.
