# Supplementary material for: Validation of the German language version of the Chronic Ear Survey and its psychometric comparison with an established German measurement instrument
Source: HNO. 2023 Oct 4;72(Suppl 1):33–42. doi: 10.1007/s00106-023-01335-5 (PMC10799145; doi:10.1007/s00106-023-01335-5)
Supplement: Supplementary file 1 [file 106_2023_1335_MOESM1_ESM.pdf]

**The Chronic Ear Survey**

Nadol, J.B., Jr., Staecker, H. and Gliklich, R.E. (2000), Outcomes Assessment for Chronic Otitis Media: The Chronic Ear Survey. The Laryngoscope, 110: 32-35. https://doi.org/10.1097/00005537-200003002-00009

Copyright © 1997 Massachusetts Eye and Ear Infirmary and Outcome Science, Llc. Reprinted with permission from J. B. Nadol. All rights reserved.

Activity Restriction Based–Subscale

a1. Because of your ear problem, you don't swim or shower without protecting your ear.

Definitely true True False Definitely false

a2. At the present time, how severe a limitation is the necessity to keep water out of your ears?

Very severe Severe Moderate Mild Very mild None

a3. In the past four weeks, has your ear problem interfered with your social activities with friends, family or groups?

All of the time Most of the time A good bit of the time Some of the time A little of the time None of the time

Symptom Subscale

s1. Your hearing loss is:

Very severe Severe Moderate Mild Very mild None

s2. Drainage from you ear is:

Very severe Severe Moderate Mild Very mild None

s3. Pain from your ear is:

Very severe Severe Moderate Mild Very mild None

s4. Odor from your ear is very bothersome to you and/or others:

Definately true True Don't know False Definately false

s5. The hearing loss in your affected ear bothers you:

All of the time Most of the time A good bit of the time Some of the time A little of the time None of the time

s6. In the past 6 months, please estimate the frequency that your affected ear has drained:

Constantly 5 or more times, but not constantly 3–4 times 1–2 times Not at all

s7. The odor from your affected ear bothers you and/or others:

All of the time Most of the time A good bit of the time Some of the time A little of the time None of the time

Medical Resource Subscale

m1. In the past 6 months, how many separate times have you visited your doctor, specifically about your ear problem?

More than 6 times 5–6 times 3–4 times 1–2 times None

m2. In the past 6 months, how many separate times have you used oral antibiotics to treat your ear infection?

More than 6 times 5–6 times 3–4 times 1–2 times None

m3. In the past 6 months, how many separate times have ear drops been necessary to treat your ear condition?

More than 6 times 5–6 times 3–4 times 1–2 times None
